# Umbilical cord blood cell characteristics in very preterm neonates for autologous cell therapy of preterm-associated complications

**DOI:** 10.1186/s12887-024-04678-2

**Published:** 2024-03-25

**Authors:** Ren Zhuxiao, Han Jiangxue, Li Yongsheng, Pei Jingjun, Yang Shuo, Xu Fang, Zhang Qi, Zhang Shandan, Nie Chuan, Yang Jie

**Affiliations:** 1 Department of Neonatology, Guangdong Women and Children Hospital, Guangzhou, 511442 China; 2 Guangdong Neonatal ICU Medical Quality Control CenterNational Key Clinical Specialty Construction Unit, Guangzhou, 511442 China; 3 Guangdong Cord Blood Bank, Guangzhou, 511440 China; 4 Department of Neonatology, Nanfang Hospital, Southern Medical University, Guangzhou, 511400 China; 5 Department of Medical Statistics, School of Public Health, Sun Yat-Sen University, Guangzhou, 510080 China; 6 Department of Clinic Genetic Center, Guangdong Women and Children Hospital, Guangzhou Medical University, Guangzhou, 511442 China; 7 Department of Neonatology, The Maternal and Child Health Care Hospital of HuaDu District, GuangZhou City, Guangdong Medical University, Guangzhou, 510800 China

**Keywords:** Cord blood cells, Feasibility, Cell therapy, Very preterm infants, Outcomes

## Abstract

**Background:**

There are emerging clinical evidence for umbilical cord blood mononuclear cells (UCBMNCs) intervention to improve preterm complications. The first critical step in cell therapy is to obtain high-quality cells. This retrospective study aimed to investigate the quantity and quality of UCBMNCs from very preterm infants (VPIs) for the purpose of autologous cell therapy in prevention and treatment of preterm complications.

**Methods:**

Very preterm infants (VPIs) born in Guangdong Women and Children Hospital from January 1, 2017, to December 8, 2022, from whom cord blood was successfully collected and separated for public or private banking, were enrolled. The UCBMNCs characters from route cord blood tests performed in cord blood bank, impact of perinatal factors on UCBMNCs, the relationship between UCBMNCs characteristics and preterm outcomes, and the correlation of UCBMNCs characteristics and peripheral blood cells in VPIs were analyzed.

**Results:**

Totally, 89 VPIs underwent UCB collection and processing successfully. The median cell number post processing was 2.6 × 10^8^. To infuse a dose of 5 × 10^7^ cells/kg, only 3.4% of infants required a volume of more than 20 mL/kg, which exceeded the maximum safe volume limit for VPIs. However, when infusing 10 × 10^7^ cells/kg, 25.8% of infants required a volume of more than 20 ml/kg volume. Antenatal glucocorticoids use and preeclampsia was associated with lower original UCBMNCs concentration. Both CD34+ hematopoietic stem cells (HSC) frequency and colony forming unit - granulocyte and macrophage (CFU-GM) number correlated negatively with gestational age (GA). UCBMNCs characters had no significant effect on preterm outcomes, whereas a significant positive correlation was observed between UCBMNCs concentration and total white blood cell, neutrophil, lymphocyte and PLT counts in peripheral blood.

**Conclusion:**

UCBMNCs collected from VPIs was feasible for autologous cell therapy in improving preterm complications. Setting the infusion dose of 5 × 10^7^ cells/kg guaranteed a safe infusion volume in more than 95% of the targeted infants. UCBMNCs characters did not affect preterm complications; however, the effect of UCBMNCs concentration on peripheral blood classification count should be considered when evaluating the immunomodulation of UCBMNCs transfusion.

## Significance statement

This retrospective observational study showed umbilical cord blood mononuclear cells (UCBMNCs) collected from VPIs was feasible for autologous cell therapy for preterm complications. Setting the infusion dose of 5 × 10^7^ cells/kg guaranteed a safe infusion volume in more than 95% of the targeted infants. UCBMNCs characteristics were not related to preterm complications; however, the effect of UCBMNCs concentration with peripheral blood classification count should be considered when evaluating the immunomodulation of UCBMNCs transfusion. These results will promote the collection of UCB in very preterm infants for autologous cell therapy, help optimize the dose for autologous treatment and lay an important foundation for the future clinical trials.

## Introduction

Preterm complications remain the primary cause of death in preterm infants [1, 2]. Increasing evidence has shown the great potential of cord blood derived cell therapy in improving preterm complications [3–9]. Cord blood-derived stem cells have several advantages, including easy extraction, low immunogenicity, and high proliferation capacity, without harming contributors [10–12]. However, cord blood collection for banking in very preterm infants (VPIs) (< 32 weeks of gestational age [GA]) is not recommended because of lower cell volume and number compared with older infants [13, 14]. Thus, valuable cord blood cells from preterm infants are usually discarded.

Several clinical studies on stem cell therapy in preterm infants have used allogenic umbilical cord blood (UCB) derived cells [6, 7, 15–17]. Compared with allogeneic cell sources, autologous UCB cells have no risk of immunological rejection and less risk of infection because of the one-step gravity gradient separation method [12, 8]. However, since there is little experience and no guidelines on the collection of cord blood cells in VPIs, and the quantity and quality of the cell products is closely related to individual samples, there are still many concerns regarding the feasibility of umbilical cord blood mononuclear cells (UCBMNCs) for autologous cell therapies in very preterm populations, who are at the highest risk of developing premature complications.

In previously reported clinical studies which used UCBMNCs to prevent and treat preterm complications, the highest cell dose chosen was 5 × 10^7^ cells/kg [8, 12]. It is increasingly recognized that immunomodulation represents an important mechanism underlying the benefits of stem cell therapies in improving premature complications [3, 4, 18–20]. The beneficial effects of cell therapy was related to the cell doses recieved [21]. However, the previous study did not provide evidence for the chosen cell doses, and whether a higher cell dose was possible to be used in clinical trials was not investigated. Furthermore, the variation in individual cord blood cell parameters may be a notable confounding factor in evaluating the effect of UCBMNCs intervention on outcomes and immunoregulatory function. Thus, it is necessary to know whether the initial UCBMNCs characters affects preterm outcomes and peripheral blood cells.

To address these questions, this retrospective observational study aimed to investigate the UCBMNCs characteristics collected from VPIs for the purpose of autologous cell therapy in preterm complications. These results will promote the collection, storage and clinical application of UCB from VPIs and help optimize the dose for autologous cell therapy for preterm complications.

## Methods

### Study design

**Fig. 1:**
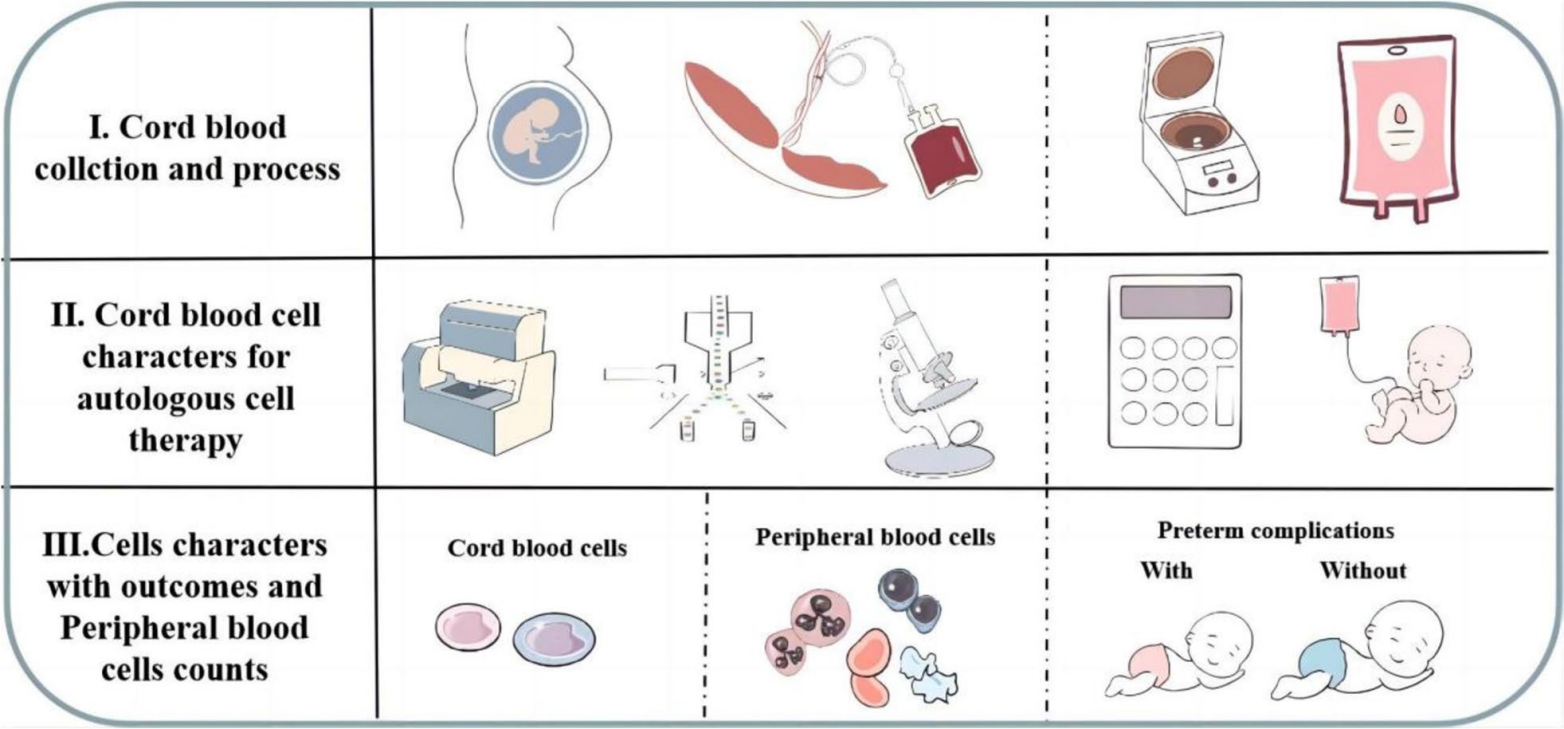
The study flow diagram

This was a retrospective observational study. The design and process are showed in Fig. 1. This study was approved by the Ethics Committee of Guangdong Women and Children Hospital (201801062). Written informed consent was obtained from the parents of all the enrolled infants.

### Study participants

VPIs born in Guangdong Women and Children Hospital from January 1, 2017, to December 8, 2022, from whom cord blood was successfully collected and separated for public or private banking, were enrolled.

### Inclusion criteria

Infants fulfilling all the following inclusion criteria were enrolled in this study: (1) born at study hospital; (2) gestational age (GA) < 32 weeks (GA was calculated based on the date of the last menstrual period of the mother and an ultrasonographic screening performed during the first trimester of pregnancy); (3) free of severe perinatal asphyxia (defined as an Apgar score of 0–3 for > 5 min, a cord blood gas pH < 7.00, or both); (4) free of severe congenital anomalies or genetic syndromes; (5) UCB was collected and processed successfully.

### Exclusion criteria

Infants were excluded from the study if (1) they exhibited severe congenital abnormalities (detected via prenatal ultrasonography or found soon after birth) or (2) their parents did not provide written informed consent.

### Enrollment and cord blood collection

Written informed consent was obtained from the parents or guardians of infants before delivery. Delayed cord clamping (DCC) for 30–60 s was routinely conducted in preterm infants that do not need immediate resuscitation after birth. UCB collection was conducted after DCC. After the neonate was born and before the placenta was delivered, umbilical cord were sterilized and the umbilical vein was punctured with a 17-gauge needle. Cord blood was collected using a blood-collection bag by the trained medical staff. The Guang Dong Cord Blood Bank (GDCBB) is a provincial, public cord blood bank, with the clinical certification of AABB (American association of blood bank) and authentication of Chinese Ministry of Health. GDCBB routinely collects cord blood in cases with consent at our hospital.

The blood-bag tubing was closed and sealed as the collection was completed. The cord blood was labeled with the full name of neonate, the hospital name and the birth time, and then placed in a cord blood storage container at 4 °C storage and sent to the Guangdong Cord Blood Bank. Since UCB in very preterm infants could not fulfill the criteria for storage in public bank. In this study, the UCB information was from the UCB sent for self storage, none of the children that underwent UCB collection ultimately used UCB for preventing these complications.

### Cord blood processing

Procedures for cord blood processing was performed in accordance with cord blood bank guidelines as described previously [5]. Briefly, after arriving GDCBB, blood bag was transported into ten thousand-grade aseptic operational workshop via transmitting window. Two milliliter(ml) cord blood was taken to detect viruses (human immunodeficiency virus, hepatitis B virus, hepatitis C virus, cytomegalovirus by polymerase Chain Reaction, PCR) and bacterial infections (including Treponema pallidum-antibody detection; or blood smear). The results were obtained before the transfusion was started. Blood bags were then centrifuged in 4 degree celsius with gravity of 50 g for 8 min in low-temperature centrifuge (RT, Beckman, USA). Then red blood cells were removed. After that, blood bags were turned straight, centrifuged for 10 min in the same condition as previous. Then MNCs were obtained after the plasma was separated by the plasma separation clip. Finally, 1ml MNCs sample was taken to have detection as the following: MNCs counts (Sysmex XE-5000 automated flow cytometer, Japan), CD34+ cell counts (BD calibur flow cytometry, BD Bioscience, USA), colony-forming unit-granulocyte and macrophage (CFU-GM) counts under inverted microscope after being cultured in methyl cellulose medium (SIGMA-ALDRICH, USA), sterility detection by automatic bacteria detector (Thermo Fisher 6240, MA, USA) and cell viability by 7-aminoactinomycin D detection kit via flow cytometry analysis.

### Clinical and cord blood cell characteristics data

Baseline perinatal characteristics of both infants and mothers, preterm complications outcome before discharge home, and cord blood cell characteristics were extracted from the electronic medical record system and entered into an online database.

1. Basic clinical characteristics included patient birth date, gender, gestational age, birth weight, delivery mode, Apgar score at 1 and 5 min, prenatal glucocorticoid usage (the indication for antenatal corticosteroid use was high risk of preterm delivery within 7 days), maternal preeclampsia, gestational hypertension and gestational diabetes.
2. The preterm outcomes included preterm complications-intraventricular hemorrhage (IVH), necrotizing enterocolitis (NEC), retinopathy of prematurity (ROP), late onset sepsis (LOS), bronchopulmonary dysplasia (BPD), respiratory distress syndrome (RDS), anemia, and mortality.

The diagnosis of common preterm complications included the following [22]:

- BPD was defined as treatment with oxygen > 21% for at least 28 days. Neonates were diagnosed by BPD severity according to the mode of respiratory support administered at a PMA of 36 and 0/7 weeks by using the diagnostic criteria proposed in 2001. No bronchopulmonary dysplasia: no support; mild BPD: nasal cannula≦2 L/min; moderate BPD: nasal cannula > 2 L/min or noninvasive positive airway pressure; and severe BPD: invasive mechanical ventilation. For neonates discharged from the hospital before 36 weeks’ PMA, BPD severity was categorized on the basis of the respiratory support administered at discharge.
- RDS was defined if the infants showed evidence of respiratory symptoms such as grunting and chest retraction, typical chest radiography findings, and/or treatment with surfactant, and the need for assisted ventilation.
- NEC was defined using Bell’s classification, and any stage of NEC was calculated in this study [23].
- LOS was defined if the infants had a positive bacterial culture results after the first 72 h after birth.
- ROP was defined according to the International Classification for Retinopathy of Prematurity [24].

Anemia was defined as hemoglobin level being less than 140mg/l.

Diagnosis of intraventricular hemorrhage (IVH) was done by cranial sonography, magnetic resonance imaging, or computed tomography during hospitalization. The first head ultrasound was performed within 3 days after birth and follow-up head ultrasound examinations was performed every 1 or 2 weeks until the day of discharge [25].

3. The characteristics of cord blood cells included cord blood volume, MNC concentration, and total number of MNCs before and after processing; number of MNCs per kg, CFU-GM number, CD34+ cell proportion, cell viability post processing; cord blood volume per kg required for infusing 1,5 and 10 × 10^7^ cells/kg, and proportion of infants who required infusion of > 20 mL/kg to reach a dose of 1, 5, and 10 × 10^7^ cells/kg. We set the maximum safe volume for UCBMNCs transfusion as 20 mL/kg each time [22].
4. The first peripheral blood cell classification and count from routine blood tests within 24 h after birth included white blood cells, platelets, neutrophils, lymphocytes counts, hemoglobin and C-reactive protein (CRP) levels.

### Perinatal factors and UCBMNCs characters

The effect of gender, gestational age, birth weight, delivery mode, Apgar score at 1 and 5 min, prenatal glucocorticoid usage, maternal preeclampsia, gestational hypertension and gestational diabetes on UCBMNCs characters were assessed using multiple linear regression analysis.

### Cord blood MNC characteristics and clinical outcomes

Differences in cord blood cell characteristics may affect preterm outcomes [26, 27]. We further analyzed the effects of cord blood MNC parameters on common preterm complications using multiple logistic regression analysis.

### Cord blood MNC characteristics and first peripheral blood cell classification and count after birth

Individual cord blood cell characteristics may affect peripheral blood cell counts and may be a notable confounding factor when evaluating the immunoregulatory effect of UCBMNCs intervention on peripheral blood. Therefore, we investigated the effect of cord blood cell parameters on peripheral blood cell classification counts using multiple linear regression analysis.

### Statistical analysis

Means, standard deviations, medians, and ranges were reported for continuous variables. Numbers and percentages were reported for categorical variables. Group comparisons of variables for cord blood parameters were performed using Student’s t-test or nonparametric tests, Fisher’s exact test, or chi-square test, as appropriate.

Multiple linear regression was used to investigate the effect of perinatal factors on cord blood cell parameters, and the cord blood cell parameters on peripheral blood cell classification counts. Then, Spearman’s correlation was used to determine the association between cord blood cell parameters and peripheral blood cell counts. Multiple logistic regression analysis was performed to estimate the effects of cord blood cell parameters on preterm outcomes. The distribution characteristics of the variables were estimated using a single-sample Kolmogorov–Smirnov test. All statistical tests were two-tailed, and statistical significance was set at *P* < 0.05. All statistical analysis were done using SPSS 21.0 (IBM).

## Results

### Study population

From January 1, 2017, to December 8, 2022, 102 VPIs born in Guangdong Women and Children Hospital, who fulfilled the enrollment criteria, consented for UCB collection and storage in Gaungdong Cord Blood Bank. 13 infants failed cord blood collection, among which five had placental abruption, five had decreased cord blood flow, and three had precipitated labor. Finally, UCB was successfully collected from 89 (87.25%) patients, all of whom had available UCBMNCs parameters and outcome data. Among them, 35 (39.3%) had a GA of < 30 weeks. The baseline clinical characteristics and incidence of common preterm complications are showed in Table 1.

**Table 1:** Characteristics and outcomes of very preterm cases with available UCB

Characters	Total (*n* = 89)
Birth weight, median, range	1450(800,2950)
GA < 30 weeks, n, (%)	35(39.3)
GA,median, range	30.43(26.57,31.86)
Male,n,%	43(48.3)
Preeclampsia,n,%	11(12.4)
Gestational hypertension,n,%	7(7.9)
Gestational diabetes,n,%	33(37.1)
Antenatal glucocorticoids, n, (%)	38(42.7)
CS, n, (%)	44(49.4)
Apgar Score	
1min, median (IQR)	9(4,9)
5min, median (IQR)	10(5,10)
IVH, n, (%)	30(33.7)
NEC, n, (%)	19(21.3)
ROP, n, (%)	18(20.2)
LOS, n, (%)	4(4.5)
BPD, n, (%)	18(20.2)
Anemia, n, (%)	48(53.9)
RDS, n, (%)	88(98.9)
Death, n, (%)	0(0.0)

*UCB* Umbilical cord blood, *GA* Gestational age, *CS* Caesarean section, *IQR* Interquartile range, *n* Number, *IVH* Intraventricular hemorrhage, *NEC* Necrotizing enterocolitis, *ROP* Retinopathy of prematurity, *LOS* Late onset sepsis, *BPD* Bronchopulmonary dysplasia, *RDS* Respiratory distress syndrome

### Characteristics of cord blood cells

**Fig. 2:**
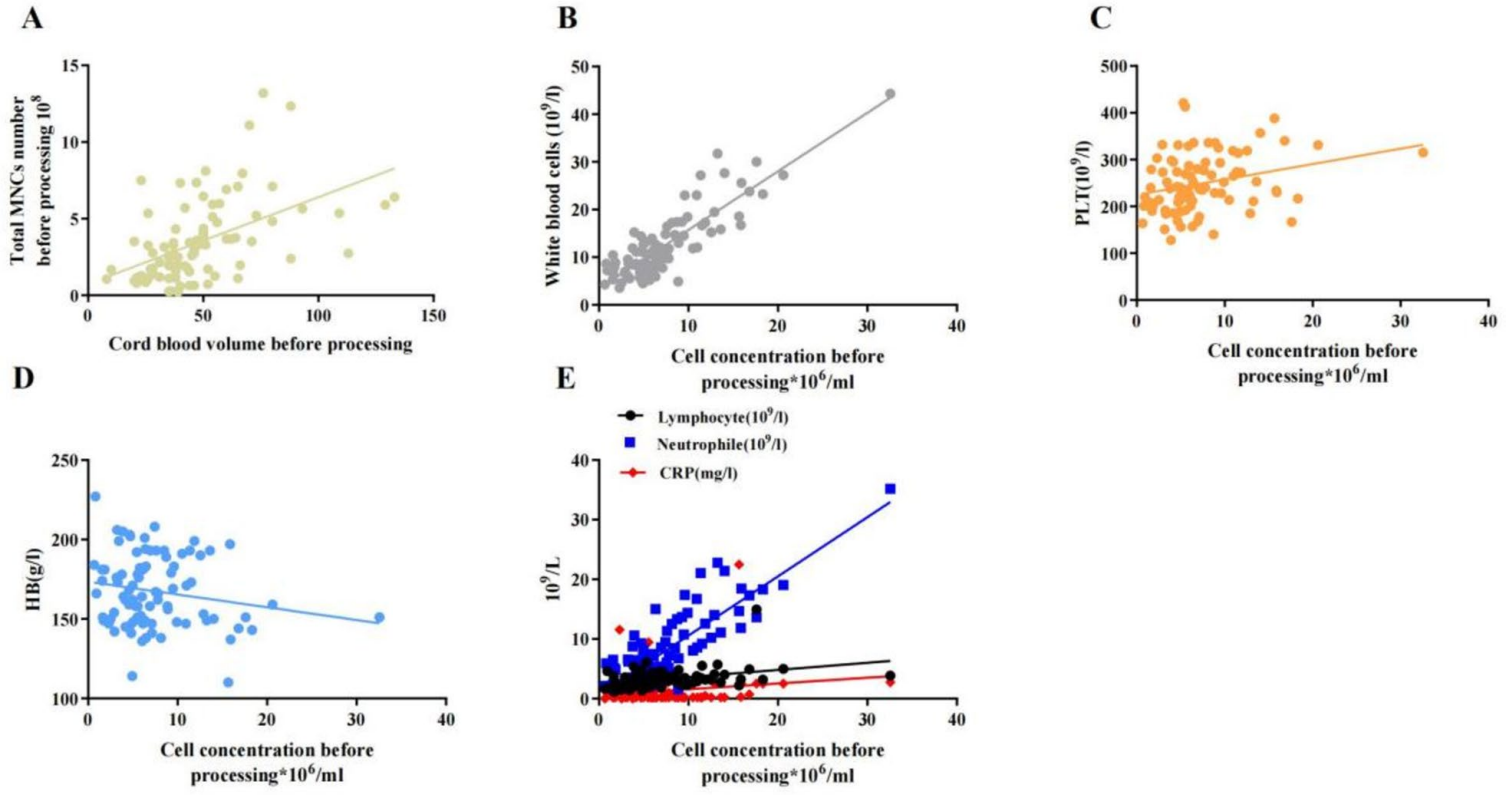
The relationship of total MNCs number and cord blood volume, and the correlation between cord blood cells concentration and the peripheral blood cells count after birth (**A** MNCs number and cord blood volume-*R* = 0.508, *p* < 0.001, **B** cord blood cells concentration and white blood cells: *R* = 0.862, *p* < 0.0001; **C** cord blood cells concentration and PLT number: *R* = 0.274, *p* = 0.010; **D** cord blood cells concentration and HB level: *R* = 0.182, *p* = 0.090; **E** cord blood cells concentration and neutrophile: *R* = 0.834, *p* < 0.0001; lymphocyte: *R* = 0.369, *p* = 0.0004; and CRP: *R* = 0.170, *p* = 0.1132)

The cord blood volume collected ranged from 8 to 133 mL (median, 44 mL); volume post processing ranged from 16 to 44 mL (median, 25 mL); cells collected ranged from 0.22 to 13.20 (× 10^8^) (median, 2.76 × 10^8^); cells post processing ranged from 0.21 to 10.93 (× 10^8^) (median, 2.6 × 10^8^); cell concentrations before processing ranged from 0.65 to 32.57 × 10^6^/mL (median 16.13 × 10^6^/mL); cell concentrations after processing ranged from 1.03 to 42.02 × 10^6^/mL (median, 9.24 × 10^6^/mL). CFU-GM ranged from 0.72 to 11.27 (/10^5^cells) (mean, 3.72 ± 3.25 × 105). The proportion of CD34 + HSC in units varied widely, ranged from 0.1% to 16.22% (median, 0.57%). The viability of post-processing units was high, ranging from 99.5 to 100% (median, 96.73%). All infants had available total cell numbers of more than 1 × 10^7^/kg; 92.1% and 67.4% had total cell numbers of more than 5 × 10^7^/kg and 10 × 10^7^/kg, respectively. All infants reached 1 × 10^7^/kg transfusion dose within a volume of less than 20 mL/kg. To reach a 5 × 10^7^/kg transfusion dose, 3.4% of infants required volumes > 20 mL/kg, whereas to reach the dose of 10 × 10^7^ cells/kg, 25.8% of patients required volumes > 20 mL/kg. The total MNCs number correlated positively with cord blood volume (*R* = 0.508, *p* < 0.001, Fig. 2A). There was no significant difference of the cord blood cell characters between infants less or more than 30 weeks of GA. Details are presented in Table 2. For the tests of viruses and bacteria in UCB, 6 infants had positive results for HBsAg, since their mothers were hepatitis B virus carrier. Two infants had positive results for bacteria, their mother had fever during delivery, but there were no chorioamnionitis. These UCB that had positive results for viruses and bacteria were excluded for storage and usage.

**Table 2:** Characteristics of the cord blood cells in very preterm infants

	Total (*n* = 89)	GA < 30 weeks (*n* = 35)	GA ≥ 30weeks (*n* = 54)	*P*
Volume collected(ml),median, range	44(8,133)	44(20,129)	41(8,133)	0.311
Volume post processing(ml),median,range	25(16,44)	25(16,30)	25(17.44)	0.239
Total cells collected (10^8^),median,range	2.76(0.22,13.20)	2.77(0.22,11.10)	2.75(0.25,13,20)	0.668
Total cells post processing (10^8^),median,range	2.6(0.21,10.93)	2.6(0.21,9.48)	2.45(0.25,10.93)	0.665
Total cells ratio (after/before),median,range	0.91(0.63,1.00)	0.91(0.80,0.99)	0.91(0.63,1.00)	0.480
Cell concentration before processing*10^6^/ml,median,range	6.13(0.65,32.57)	6.04(0.65,18.33)	6.23(0.82,32.57)	0.775
Cell concentration post processing*10^6^/ml,median,range	9.24(1.03,42.02)	10.85(1.03,32.70)	.96(1.39,42.02)	0.486
Cell number (10^7^)/kg post processing,median,range	15.79(1.52,90.29)	21.98(1.81,90.29)	13.77(1.52,88.75)	0.114
Cell number < 5 × 10^7^/kg,n,%	7(7.9)	2(5.7)	5(9.3)	0.757
Cell number 5-9.99 × 10^7^/kg,n,%	22(24.7)	8(22.9)	14(25.9)	
Cell number ≥ 10 × 10^7^/kg,n,%	60(67.4)	25(71.4)	35(64.8)	
Volumes/kg required for infusing 1 × 10^7^ cells/kg,median,range	1.08(0.24,9.71)	0.92(0.31,9.71)	1.12(0.24,7.19)	0.486
Infused volumes > 20ml/kg required for 1 × 10^7^ cells/kg,n,%	0%	0%	0%	1.000
Volumes/kg required for infusing 5 × 10^7^ cells/kg,median,range	5.42(1.19,48.54)	4.61(1.53,48.54)	5.58(1.19,35.97)	0.486
Infused volumes > 20ml/kg required for 5 × 10^7^ cells/kg,n,%	3(3.4)	2(5.7)	1(1.9)	0.559
Volumes/kg required for infusing 10 × 10^7^ cells/kg,median,range	10.82(2.38,97.09)	0.22(3.05,97.09)	11.16(2.38,71.94)	0.486
Infused volumes > 20ml/kg required for 10 × 10^7^ cells/kg,n,%	23(25.8)	9(25.7)	14(25.9)	1.000
Cell viability after processing(%),median, range	96.73(90.37,98.79)	96.74(90.37,98.54)	96.51(90.47,98.79)	1.000
CD34 + cells proportion after processing(%),median, range	0.57(0.09,1.87)	0.66(0.15,1.50)	0.51(0.09,1.87)	0.225
CFU-GM number/10^5^MNCs,median, range	7.33(0.21,241)	7.75(0.91,219)	6.8(0.21.241)	0.480

*GA* Gestational age, *MNCs* Mononuclear cells, *CFU-GM* Colony forming unit -granulocyte and macrophage, *CD34* Cluster differentiation 34

### Perinatal factors and cord blood cell parameters

Among the perinatal factors analyzed in our study, only antenatal glucocorticoids, preeclampsia and GA influenced the UCBMNCs characters. Antenatal glucocorticoids (*P* = 0.005,*OR*:-3.144) and preeclampsia (*P* = 0.007,*OR:*-5.112) were associated with lower cell concentration, and antenatal glucocorticoids was also related with more CFU-GM (*P* = 0.000,*OR*:87.243). More immature infants had higher CD34+ HSC cells (*P* = 0.030,*OR*:-0.057) and CFU-GM concentration (*P* = 0.032,*OR*:-9.838) (Table 3). No other perinatal factors were associated with cord blood cell parameters.

**Table 3:** Multiple linear regression analysis for the perinatal factors affecting cord blood cells characters

Cord blood cells characteristics	Cell concentration before processing*10^6^/ml	Cord blood volume, ml	Cell viability,%	CD34% in MNCs	CFU-GM number/10^5^ MNCs
	*P* Value, *OR* (95% CI)				
Antenatal glucocorticoids	***0.005,-3.144(-5.312,-0.976)***	0.493,-4.382,(-14.886,7.231)	0.073,-0.877(-1.837,0.083)	0.076,0.132(-0.014,0.277)	***0.000,87.243(61.933,112.493)***
Preeclampsia	***0.007,-5.112(-8.781,-1.442)***	0.954,0.546(-18.169,19.260)	0.512,-0.537(-2.162,1.088)	0.373,-0.111(-0.358,0.136)	0.188,-28.481(-71.213,14.251)
GA(weeks)	0.382,0.339(-0.429,1.108)	0.822,0.445(-3.475,4.365)	0.376,-0.152(-0.492,0.188)	***0.030,-0.057(-0.109,-0.006)***	***0.032,-9.838(-18.789,-0.887)***

*CS* Caesarean section, *GA* Gestational age, *kg* Kilogram, *CI* Confidence interval, *OR* Odds ratio, *CFU-GM* Colony forming unit -granulocyte and macrophage, *CD34* Cluster differentiation 34, *MNCs* Mononuclear cells, *OR* Odds ratio

### Cord blood cell parameters and outcomes

 When we investigated the effect of autologous cord blood stem cell therapy, the individual cord blood cell parameters itself might have affected the outcomes. Therefore, it was necessary to explore the relationship between the cell parameters and the outcomes. We found that none of the cells characters including original cell concentration, cord blood volume before processing, CD34+ cells frequency, cell viability and CFU-GM concentration affected preterm complications after adjusted for antenatal glucocorticoids, preeclampsia and GA that influenced the UCBMNCs characters (Table 4).

**Table 4:** The correlation of UCBMNCs characters with very preterm outcomes and peripheral blood differential count day one after birth

UCBMNCs characters	Cell concentration before processing*10^6^/ml	CD34% in MNCs	Cell viability,%	Cord blood volume before processing, ml	CFU-GM number/10^5^ MNCs
BPD					
* P*	0.25	0.274	0.721	0.98	0.359
* OR, 95% CI*	1.056,0.963–1.158	0.389(0.072,2.112)	1.045(0.821,1.330)	1(0.979,1.002)	0.996(0.998,1.004)
NEC					
* P*	0.565	0.311	0.904	0.198	0.319
* OR, 95% CI*	1.025,0.943–1.114	2.315(0.492,9.256)	0.986(0.786,1.003)	0.983(0.958,1.009)	1.003(0.997,1.010)
LOS					
* P*	0.203	0.561	0.051	0.784	0.238
* OR, 95% CI*	0.788,0.546–1.137	1.27(0.567,2.843)	0.567(0.321,0.883)	1.005(0.967,1.046)	1.007(0.995,1.020)
IVH					
* P*	0.565	0.072	0.390	0.736	0.189
* OR, 95% CI*	1.025,0.943,1.114	3.529(0.893,13,943)	0.918(0.756,1.116)	0.997(0.978,1.016)	1.004(0.998,1.010)
ROP					
* P*	0.411	0.231	0.424	0.124	0.060
* OR, 95% CI*	0.952,0.847–1.070	2.501(0.559.11.191)	1.109(0.860,1.431)	1.016(0.996,1.037)	1.006(1.000,1.013)
RDS					
* P*	0.159	0.252	0.986	0.893	0.672
* OR, 95% CI*	0.862,0.700–1.060	0.034(0.000,11.014)	0.992(0.404,2.433)	0.995(0.922,1.074)	1.010(0.963,1.061)
Anemia					
* P*	0.366	0.164	0.374	0.598	0.161
* OR, 95% CI*	1.042,0.953–1.139	2.997(0.639,14.063)	0.914(0.749,1.115)	1.005(0.987,1.024)	1.005(0.998,1.011)
White blood cells (10^9^/l)					
* P, R*	***0.000,0.862***				
* OR, 95% CI*	***1.229,1.073–1.385***				
Neutrophile(10^9^/l)					
* P, R*	***0.000,0.834***				
* OR, 95% CI*	***0.992,0.851–1.134***				
Lymphocyte(10^9^/l)					
* P, R*	***0.000,0.369***				
* OR, 95% CI*	***0.119,0.055–0.184***				
PLT(10^9^/l)					
* P, R*	***0.010,0.273***				
* OR, 95% CI*	***3.294,0.791–5.776***				
HB(g/l)					
* P, R*	0.090,0.183				
* OR, 95% CI*	-0.803,-1.736–0.129				
CRP(mg/l)					
* P, R*	0.113.0.171				
* OR, 95% CI*	0.096,-0.023–0.216				

*UCBMNCs* Umbilical cord blood mononuclear cells, *IVH* Intraventricular hemorrhage, *NEC* Necrotizing enterocolitis, *ROP* Retinopathy of prematurity, *LOS* Late onset sepsis, *BPD* Bronchopulmonary dysplasia, *RDS* Respiratory distress syndrome, *PLT* Platelet, *HB* Hemoglobin, *CRP* C-reactive protein, *CI* Confidence interval

### Cord blood cell parameters and peripheral blood cells count

Immunomodulation is increasingly recognized as an important mechanism underlying the benefits of stem cell therapies [5, 3, 19]; however, the original cord blood cell parameters may also affect the peripheral blood cells after birth [28, 29]. We found strong positive correlations between the cord blood cell concentration before processing and white blood cell count (*p* < 0.001, *R* = 0.862), neutrophil count (*p* < 0.001, *R* = 0.834), lymphocyte count (*p* < 0.001, *R* = 0.369), and platelet number (*p* = 0.010, *R* = 0.273) after adjusted for antenatal glucocorticoids, preeclampsia and GA that influenced the MNCs characters (Table 3 and Fig. 2B-E). There was no significant correlation between cord blood volume before processing, CD34+ HSC frequency, cell viability or CFU-GM concentration and peripheral blood cell count (Table 4).

## Discussion

With rapid advances in technology in the NICU, the survival rate of preterm infants is gradually increasing. However, the short and long term adverse outcomes associated with preterm complications did not improve [30, 31]. Until now, there has been a lack of effective interventions for the complex preterm complications. Recently, several studies have shown the potential of using UCBMNCs as a new systemic and multi-organ targeted therapy for preterm complications [12, 8, 27]. Human UCBMNCs are abundant in stem cells [32, 33]. The first critical step in cell therapy is to collect and separate high-quality cells [5, 12]. Several clinical trials have demonstrated the safety of cord blood collection in preterm infants [5, 8, 12, 27]. However, gaps remain regarding important issues that affect the application of UCBMNCs in autologous cell therapy for preterm complications. We here presented the largest study analyzing the UCB units characters collected from very preterm deliveries. The results of this study will contribute to filling these following gaps.

### Cell doses selection within a safe infusion volume

Stem and precursor cells mainly exist in the UCBMNCs layer. It is considered as the most effective component of cord blood [5, 32, 33]. To achieve more UCBMNCs in a lower volume, previous studies used volume- and RBC-reduced cord blood cells to decrease the burden on the heart [5, 8, 12]. However, there was concern that UCB collection may have an impact on the transfusion of cord blood to the preterm newborns. Actually, in our center, delayed cord clamping is routinely conducted in preterm infants that do not need immediate resuscitation after birth [34]. UCB collection was performed after DCC, even we found that DCC may reduce the total volume and cell number we could obtain finally [34]. Therefore, UCB collection after DCC have no impact on the transfusion of cord blood to the newborn. On the contrary, for those infants that were not proper for DCC due to emergent events after birth, UCB collection could save valuable UCB cells for them. Considering the vulnerable heart function of VPIs, they could only accept a limited volume of infusion of < 20 mL/kg each time [22]. Therefore, the first step was to evaluate the available cell number in a safe volume. Several previous studies have used cell dosages ranging from 1 to 5 × 10^7^ cell/kg [5, 8, 12]. Our previous study showed that, to reach a targeted cell dose of 5 × 10^7^ cells/kg, the administration volume was as high as 19 mL/kg, which was only tested in 29 VPIs [5]. Machaliński et al. demonstrated that whole autologous cord blood infusion (15 mL/kg) within 48 h of birth was feasible and safe [9]. Kurtzberg et al. demonstrated that a single intravenous autologous infusion of more than 2 × 10^7^ total nucleated cells/kg could improve the cognitive outcomes of children with cerebral palsy in a dose dependent effect [21]. Because the protective effect of UCBMNCs may depend on a sufficient cell dosage, we should aim to deliver more cells within a safe volume. The current study showed that the one-step separation method preserved a median of 91% of the collected cells, with > 90% viability. All infants could obtain at least 1 × 10^7^ total nucleated cells (TNCs) per kg, within a safe volume of 20 mL/kg. It was also feasible for more than 95% infants to reach a dosage of 5 × 10^7^ TNCs per kg; however, reaching 10 × 10^7^ TNCs per kg would need to infuse 25.8% of infants at a volume of more than 20 mL/kg. Thus, it was indicated that, in future trials, selecting a targeted dose of 5 × 10^7^ TNCs per kg would be appropriate for most of the targeted VPIs. In contrast, a higher dose, such as 10 × 10^7^cells per kg was also desired in a subgroup with higher cell numbers to assess the outcomes treated with a higher cell dosage. Since the number of cord blood cells correlated with the collected blood volume positively, further increasing the collected volume without affecting patient safety was warranted. To administer multiple smaller infusions to overcome the limitations of infusion volume based on the required number of cells for therapy was also condidered. However, on the other hand, we may also consider several other issues, such as, too much volume may also cause fluid overload of the infants even infused for several times, and multiple infusions may cause higher probability of infection, in addition, longer storage of the cells may also lower the cell viability. Therefore, in clinical practice, this may also be one of the important concern. But we could try multiple smaller infusions in the future practice, too.

### Impact of perinatal factors on quality of UCB cells

Previous reports showed influence of obstetric and neonatal factors on the volume and haematopoietic content of UCB donations, however, rare study investigated the impact of perinatal factors on cord blood cell parameters in very preterm infants. In this study, we found there was no strong correlation between cord blood volume and cell concentration with GA in VPIs, interestingly, more immature infants had higher CD34+ HSC cells and CFU-GM concentration. This indicates the feasibility and great value of cord blood collection from immature infants for cell therapy in premature associated complications. This study indicated antenatal glucocorticoids and preeclampsia diminished cord blood cell concentrations, but antenatal glucocorticoids also increased CFU-GM number. To reducing RDS severity, antenatal glucocorticoids usage was recommended in VPIs, this may affect the cell quantity. In addition, obstetric strategies to prevent preeclampsia may be helpful in obtaining more MNCs. The possible explanation for antenatal corticosteroids lowering UCB concentration might be the immunosuppression function of corticosteroids, since the mononuclear cells in UCB were mostly immune cells. More research was needed to investigate underlying mechanisms.

### Cord blood cell characteristics and preterm outcomes

Previous studies have suggested a correlation between the cord blood precursor cell characteristics and preterm complications [26]. Thus, when evaluating the effects of MNCs intervention on preterm outcomes, the parameters of individual cord blood samples may be confounding factors. Therefore, we evaluated the relationship of MNCs cells characters with several common preterm complications and found no significant effect on the outcomes. Since currently the total nucleated cell content per kilogram is the main parameter used for autologous cell therapy. We hypothesized that the beneficial effects of MNCs intervention on premature-associated outcomes would be independent of the original individual MNCs parameters.

### Cord blood cell characteristics and peripheral blood cells

Several studies have demonstrated the immunomodulatory reprogramming of circulating immune cells in stem cell therapy [16, 19]. Our previous non-randomized study revealed an increased proportion of CD4 + T cells and Tregs in the cord blood MNCs intervention group [5]. Kim et al. demonstrated strong relationships between white blood cell counts and neutrophil counts in UCB and peripheral blood (PB); however, not in the absolute counts of lymphocytes, which was conducted in only 14 term patients [28]. In this current study, we showed that the cell concentration in cord blood correlated positively with white blood cell, neutrophil, lymphocyte, and platelet numbers in the first peripheral routine blood test after birth, which was not observed in other cord blood cell parameters. Therefore, when evaluating the immunomodulatory effect of UCBMNCs infusion, to distinguish whether the effect on immune cells were due to cell therapy or just proof-of-principle in nature only, the balance of individual cord blood cell concentrations in the control and intervention groups should be assessed and corrected as confounding factors if not balanced.

### Limitations

The current study had some limitations. First, extremely preterm infants (EPIs) younger than 28 weeks were at the highest risk of severe preterm complications; however, only 10 EPIs were enrolled in this study. Actually, in clinical setting, UCB of extremely preterm infants (EPI) was rarely collected for storage, because of the limited cell number and volume. More information for this subgroup would be provided in the future by performing a prospective multi-center study. Second, when evaluating the correlations between cord blood cell concentrations and peripheral blood characteristics, only routine blood test results were available, and no detailed lymphocyte typing was performed. Thus, the effect of cord blood cell concentration on lymphocyte subsets was not assessed. In addition, due to the retrospective character of this study, the levels of inflammatory factors were not measured. The levels of inflammatory factors should be measured in the future prospective study. Thirdly, although our previous study showed there was no significant difference between collecting cord blood after birth and not collecting cord blood preterm groups in terms of postnatal hemoglobin levels, hematocrit, and occurrence of complications in preterm infants less than 35 gestational weeks [34], a case-matched study to compare these parameters in very preterm infants were also needed.

## Conclusion

In conclusion, this study showed cord blood cells collected from VPIs were feasible for use in autologous cell therapy for preterm complications. Setting the targeted infusion dose to 5 × 10^7^ cells/kg guarantees a safe infused volume in most VPIs. Cord blood MNCs parameters were unrelated to preterm complications; however, the effect of individual cord blood cell concentrations on peripheral blood immune cells should be considered when evaluating the immunomodulation of UCBMNCs intervention.

## Data Availability

No datasets were generated or analysed during the current study.

## Abbreviations

UCBMNCs: Umbilical cord blood mononuclear cells
VPIs: Very preterm infants
HSC: Hematopoietic stem cells
CFU-GM: Colony forming unit - granulocyte and macrophage
GA: Gestational age
UCB: Umbilical cord blood
GDCBB: Guang Dong Cord Blood Bank
ml: Milliliter
IVH: Intraventricular hemorrhage
NEC: Necrotizing enterocolitis
ROP: Retinopathy of prematurity
LOS: Late onset sepsis
BPD: Bronchopulmonary dysplasia
RDS: Respiratory distress syndrome
CRP: C-reactive protein
PB: Peripheral blood
EPIs: Extremely preterm infants
